# Novel *COL9A3* mutation in a family diagnosed with multiple epiphyseal dysplasia: a case report

**DOI:** 10.1186/1471-2474-15-371

**Published:** 2014-11-08

**Authors:** Changhoon Jeong, Jae Young Lee, Jiyeon Kim, Hyojin Chae, Hae-il Park, Myungshin Kim, Ok-Hwa Kim, Paul Kim, Young Kee Lee, Jongsun Jung

**Affiliations:** Department of Orthopaedic Surgery, Bucheon St. Mary’s Hospital, The Catholic University of Korea, Seoul, South Korea; Catholic Genetic Laboratory Center, Seoul St. Mary’ Hospital, The Catholic University of Korea, Seoul, South Korea; Department of Laboratory Medicine, The Catholic University of Korea, Seoul, South Korea; Department of Radiology, Ajou University Hospital, Suwon, South Korea; Syntekabio Inc, Seoul, South Korea

**Keywords:** Multiple epiphyseal dysplasia, COL9A3, Molecular dynamics simulation

## Abstract

**Background:**

Multiple epiphyseal dysplasia is a common skeletal dysplasia characterized by mild short stature, early-onset osteoarthritis mainly involving the hip and knee joints, and abnormally small and/or irregular epiphyses. Multiple epiphyseal dysplasia is clinically and genetically heterogeneous and six genes are associated with the phenotype of multiple epiphyseal dysplasia.

**Case presentation:**

A 12-year-old Korean boy presented with intermittent knee pain. His height was 144.6 cm (20th percentile) and family history was notable for early-onset osteoarthritis in his father. The proband’s x-rays revealed epiphyseal changes characteristic of multiple epiphyseal dysplasia associated with a collagen IX defect, with manifestations primarily restricted to the knees. Mutational analysis identified a novel c.104G > A substitution in exon 2 of *COL9A3*, resulting in p.Gly35Asp in the proband and his father. In silico analyses predicted the p.Gly35Asp amino acid change to be detelerious, and molecular dynamics simulation demonstrated a major structural change in the heterotrimeric collagen IX.

**Conclusion:**

So far, three *COL9A3* mutations, have been reported. These three mutations are located at the splice donor or acceptor site of *COL9A3* and cause skipping of exon 3, resulting in the deletion of 12 aminoacids in the COL3 domain of COL9A3. In contrast, the novel missense mutation identified in this two-generation family with multiple epiphyseal dysplasia is a missense mutation affecting the Gly residue of the Pro-Pro-Gly repeat sequence in the COL3 domain of collage IX, with accompanying major structural change of the collagen peptide.

**Electronic supplementary material:**

The online version of this article (doi:10.1186/1471-2474-15-371) contains supplementary material, which is available to authorized users.

## Background

Multiple epiphyseal dysplasia (MED) is clinically and genetically heterogeneous group of skeletal dysplasias characterized by early-onset osteoarthritis, a waddling gait, restriction of joint mobility, pain and stiffness in the weight-bearing joints and sometimes short stature. Mutations in six different genes cause MED. Mutations in genes encoding cartilage oligomeric matrix protein (*COMP*), matrilin-3 (*MATN3*), and the alpha 1–3 chains of type IX collagen (*COL9A1*, *COL9A2*, *COL9A3*) result in autosomal dominant MED [1], and a specific mutation in the diastrophic dysplasia sulfate transporter (*DTDST*) is associated with an autosomal recessive form of MED [2]. The frequencies of mutations in these genes among MED patients have yet to be determined. Previous studies in European MED patients have shown frequencies of 7–35% for *COMP*, 14% for *DTDST*, 5–10% for *MATN3*, and 5–15% for the type IX collagen genes [3, 4]. The frequencies of causative genes showed racial differences. A comprehensive screening in a Japanese population indicated that frequencies differ in East Asian patients, with reported frequencies of 23% in *MATN3*, 20% in *COMP*, 6% in *COL9A2*, and none in *DTDST*[5]. In Korean patients, *MATN3* mutations were found in 55% of patients, followed by *COMP* mutations in 41%, and *COL9A2* and *DTDST* mutations in one patients (2%), respectively [6]. Genotype-phenotype correlations of MED has been described and MED resulting from collagen IX defects (*COL9*-MED) result in more severe involvement of the knees with relative sparing of the hips.

Here we report a two-generation family with MED phenotypes consistent with *COL9*-MED, that were caused by a novel missense mutation in *COL9A3*.

## Case presentation

### Patient’s characteristics

The proband was a 12-year-old Korean boy born to non-consanguineous parents. He had been treated for partial seizure disorder for 2 years. He was referred to the pediatric orthopedic clinic for the evaluation of intermittent knee pain that had occurred for a few months. His height was 144.6 cm (20th percentile) and bodyweight was 41 kg (60th percentile). The height of his father and mother was 170 cm and 160 cm, respectively. His father also suffered from intermittent mild knee joint pain. The proband and his father did not show muscle weakness and pain suggesting myopathy. There was no Gower sign and Trendelenberg sign on physical examination. The level of creatine kinase and lactate were normal. Informed consent was obtained from the parents of the child.

### Orthopedic and radiologic evaluations

**Figure 1:**
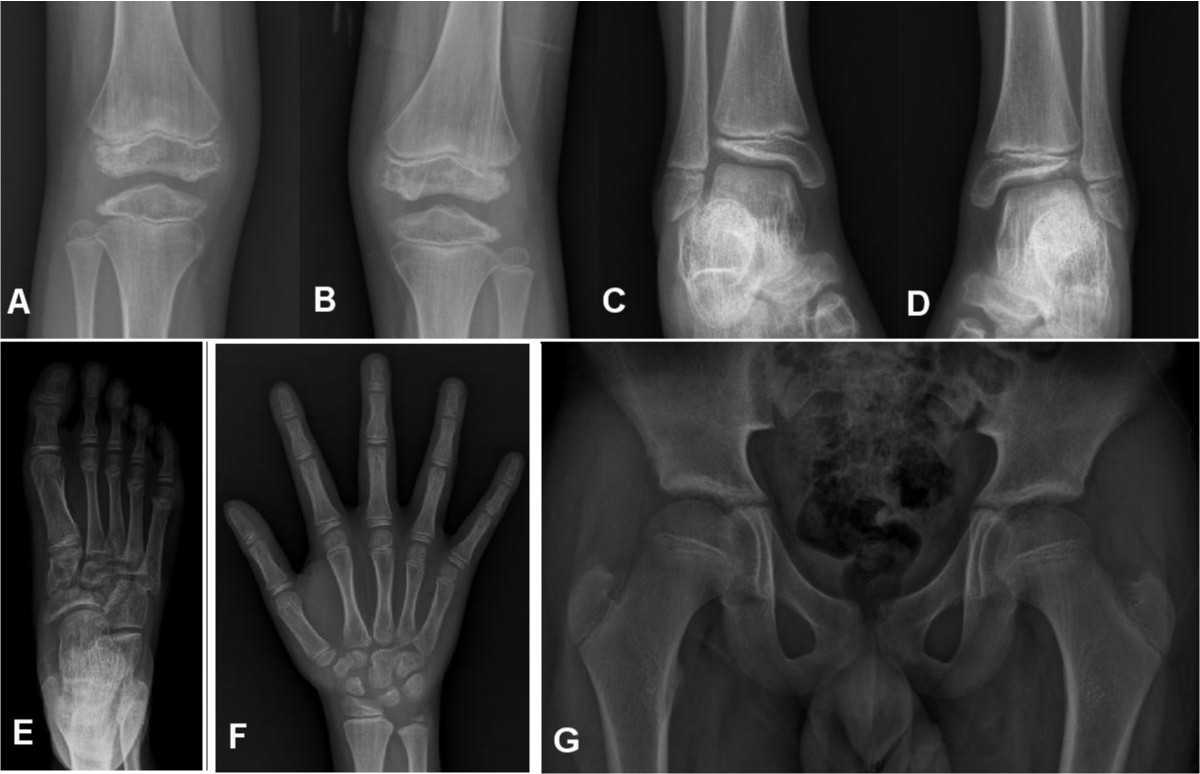
**Radiographs of proband at the age of 12 years.** Anteroposterior view of knee joint showed shortening, fragmentation, and joint surface irregularity of distal femoral and proximal tibial epiphysis **(A, B)**. Anteroposterior view of distal tibial epiphysis showed a lateral wedging and fragmentation **(C, D)**. The tarsal navicular and cuneiform bone showed irregular ossification **(E)**. The epiphysis of distal radius was wedge shaped and the epiphyses of the distal ulnae were relatively small and the carpal bone shows dysplasia and flattening **(F)**. The epiphyses of proximal femur were spared **(G)**.

**Figure 2:**
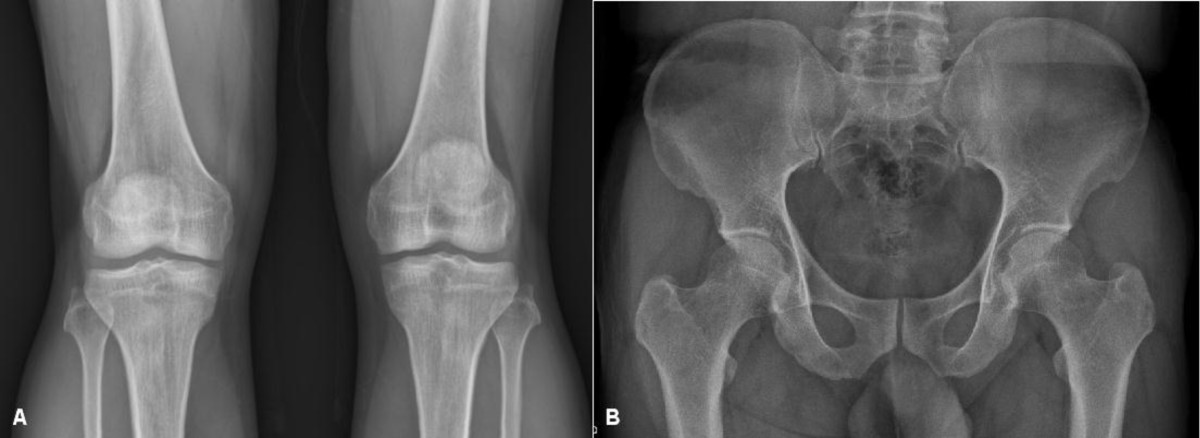
**Radiographs of the proband’s father at the age of 41 years.** Anteroposterior view of knee joints revealed early osteoarthritis with findings including of medial joint space narrowing and joint surface irregularity **(A)**. The capital femoral epiphysis of the proband’s father has no evidence of dysplasia **(B)**.

Clinically, the lower extremity of the proband showed mild genu valgum deformity. The knee joints displayed full range of motion. The distal femoral and proximal tibial epiphyses were affected most, and the distal tibia and distal radius epiphyses were affected less severely. There was no irregularity on the femoral head epiphysis. Distal femoral and proximal tibial epiphyses showed loss of height, joint surface irregularities and fragmentation (Figure 1A and B). The epiphysis of the distal tibia showed lateral shortening and fragmentation (Figure 1C and D). There was no abnormality of the distal fibular epiphysis nor the talar dome. The tarsal navicular and cuneiform bone showed irregular ossification (Figure 1E). The epiphysis of distal radius was wedged shape and the epiphyses of the distal ulnae were relatively small. The carpal bone shows dysplasia and flattening (Figure 1F). The spine and the proximal femoral epiphyses appeared normal (Figure 1G). X-rays of the proband’s father revealed osteoarthritis of Kellgren-Lawrence grade I particularly on the medial side (Figure 2A). However, the proximal femoral epiphyses were spared (Figure 2B). Other members of the family were not affected.

### Genetic analyses

PCR from genomic DNA followed by direct sequencing was performed following parental informed consent. Genomic DNA was isolated from peripheral blood leukocytes. Direct DNA sequencing of the *COMP, MANT3, COL9A1*, *COL9A2,* and *COL9A3* genes was performed. All exons were amplified using the primers previously described (Table 1). PCR amplicons were bidirectionally sequenced with the Big Dye terminator v3.1 cycle sequencing kit (Applied Biosystems, Foster City, CA) using an ABI PRISM 3130 Genetic Analyzer (Applied Biosystems). *COMP, MATN3, COL9A1* and *COL9A2* revealed no mutations. A novel missense mutation c.104G>A in exon2 of *COL9A3*, which resulted in p.Gly35Asp, was identified. RefSeq ID: NM_001853.3 reference sequence was used for cDNA nucleotide numbering. This mutation was predicted to be not tolerated by SIFT and probably damaging by PolyPhen [7, 8]. Also, comparative evolutionary analysis showed that the glycine residue at codon 35 is a highly conserved amino acid during evolution. And the same mutation was observed in the proband’s symptomatic father. We performed Sanger sequencing of 100 healthy controls and none harbored a c.104G>A mutation in exon 2 of *COL9A3.*

**Table 1:** **PCR primer sequences for** ***COL9A1, COL9A2, COL9A3, COMP,*** **and** ***MATN3***

Primer sequences			
***COL9A1*** gene		Tm (°C)	Product size (bp)
Exon 8-10	F - CCGATGTGCTCCACTAACCT	56	824
	R - GGCCAAGTTTAGAGCCACAG		
*COL9A2* gene			
Exon 2	F - CAGCTTCCTGCACTGTCTGA	56	241
	R - GACGAGGGGCACTACATCTC		
Exon 3-4	F - TGAGCCGTAGTGTGCTGTCT	56	278
	R - CTGGAGGTCAATTGGCAGAG		
*COL9A3* gene			
Exon 2	F - TTTGGGTCTCACCGAGGA	56	293
	R - GCCTGGTTTTCTCTCCATCA		
Exon 3	F - CTTGAGGGACCCCTGATTTT	56	152
	R - TGTTCTGAGTTCCCCCTTTC		
Exon 4	F - GCATTTTGCTTCATTGCTGA	56	222
	R - AATTAGGGCCGGACTCCTC		
*COMP* gene			
Exon 8-9	F - TTGAGGCGGGGTTGGGTG	64	413
	R - ACCGTGCCGAGCCGTAGAT		
Exon 10	F - AGGAGTGTGACCTTTGCCTTCT	64	334
	R - CTAGTCCAGCTTACCCCATCC		
Exon 11-12	F - GAAGTCATTCTGGCCTGGTC	64	518
	R - AGCGTTTTGTCAAAGGCTACC		
Exon 13	F - CGGGTAGCCTTTGACAAAACG	62	331
	R - GCCCGCCCACCGTAGAC		
Exon 14-15	F - GGCGGGCCCTGACTTTAG	64	546
	R - ATAACCCCGCCCCTCTGT		
Exon 16	F - GTTCTGGGTGCCAGGTTC	64	335
	R - AAGGGTTTTACGGAGGGTCAT		
Exon 17	F - TGCTCCCAACTGTCTCTCCA	64	312
	R - ACCTGGGCCTGTGTGTCC		
Exon 18-19	F - TCTGAGAGGGAAGGGTCTGG	64	443
	R - CCCTTCTCACTTCCCCCTCA		
*MATN3* gene			
Exon 3	F - AAAGGAGCCCAGAGAGCAAT	59	290
	R - CAGTCCAAAACCTGGAGCAT		

### Molecular Dynamics (MD) simulation of wild type and mutant model structure of trimer collagen IX

**Figure 3:**
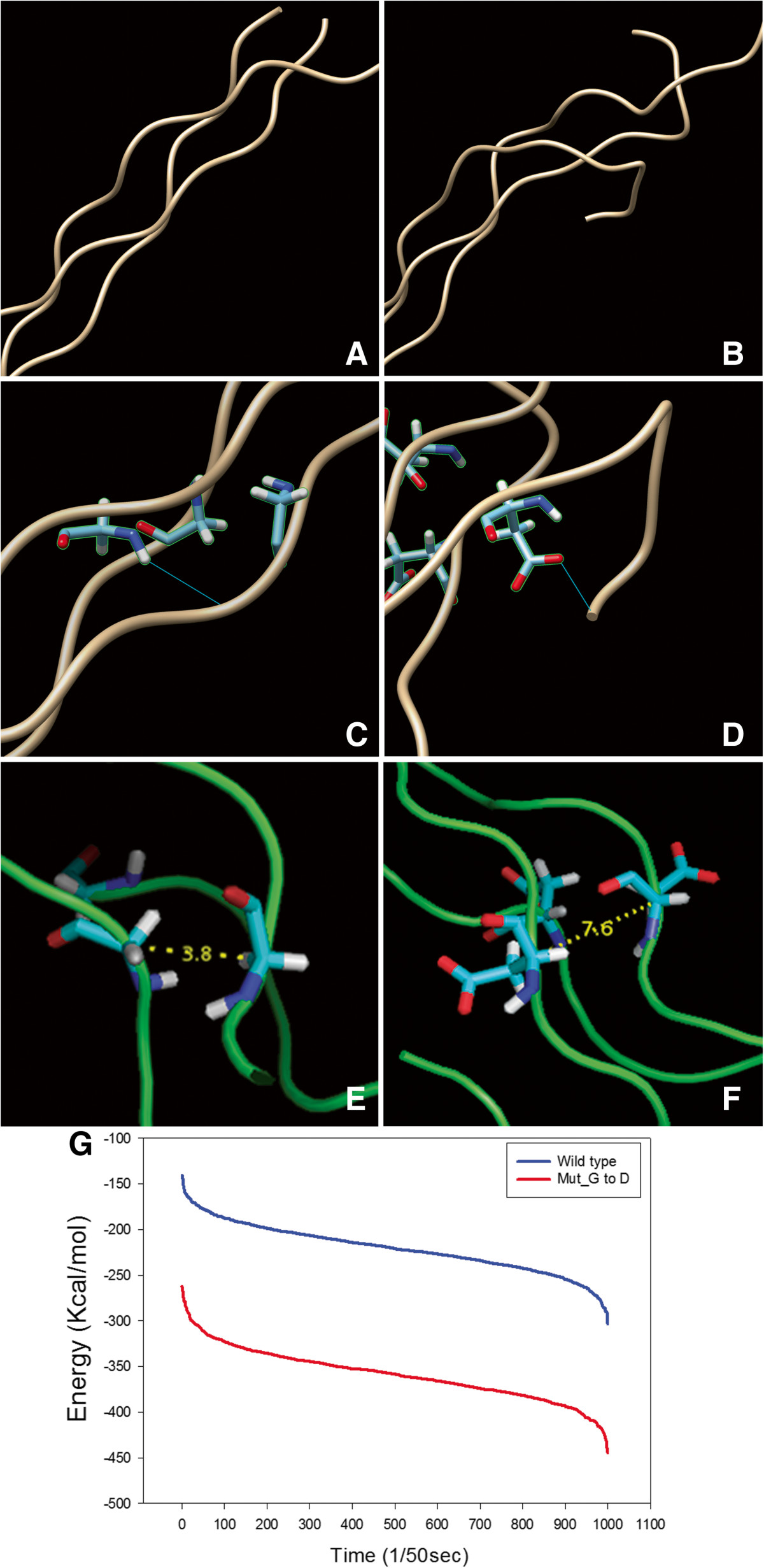
**MD simulation of wild type and mutant model structure of trimer collagen.** Wild type model structure **(A)**, twisted form of the mutant **(B)**, collagen-collagen interaction by a hydrogen bonding in wild type **(C)**, the substitution of glycine to aspartic acid causing an interaction with the self strand **(D)**, C-alpha distance between residues in wild type and mutant trimer **(E and F)**, respectively, and energy plot of wild type and mutant collagen by Amber-GPU **(G)**.

Molecular mechanics potential energy minimization and MD simulations were carried out using the program package AMBER 11 [9]. FF99 force field in AMBER package was used in all MD simulations including hetero atoms. Topology file GAFF force field in AMBER package was used. The generalized Born solvation model was used instead of explicit water [10]. The temperature was kept constant by using Langevin dynamics with a collision frequency of 5.0 ps^−1^[11]. The minimized system was heated from 0 K to 325 K for 50 ps in a total of 7 stages. By heating gradually in stages, the possible crashes will be reduced by allowing it to equilibrate at each temperature. The heated systems were then subjected to molecular dynamics simulations for 1 ns each at 325 K in a total of 10 stages. In all simulations, the temperature was kept constant at 325 K. The simulations were performed using the PMEMD module with GPU system which utilizes the particle mesh Ewald method [12]. SHAKE algorithm was used to constrain bond lengths involving hydrogen [13].The mutation of p.Gly35Asp in type IX collagen alpha 3 has a Pro-Pro-Gly consecutive residue pattern. The collagen model structure was generated with a template of 2v53 PDB (protein database bank). After the MD simulation, the wild type and mutant collagen structures were visually inspected. The wild type structure formed hydrogen bonds with its neighboring strand while the mutant was aggregated by themselves (Figure 3A to D). In addition, the distances between the mutation site (C-alpha) of the wild type and the mutant collagen are almost twice (Figure 3E and F). The calculated energy state of the mutant is much lower, causing a self aggregation (Figure 3G). Considering the major structural change caused by the collagen mutant, a malfunction of the type IX collagen is expected.

## Conclusions

The clinical and radiographic phenotypes of MED vary heterogenously according to the genetic mutation. *COMP* and *MATN3* mutations show marked abnormalities in hip and knee joints. Patients with the *COMP* mutation have characteristics of marked alterations of hip joints leading to severe osteoarthritis in early childhood [14]. The *MATN3* mutation has dysplastic epiphysis of the proximal and distal femur [15]. Clinical features of autosomal recessive MED associated with *DTDST* mutations include scoliosis and abnormal findings such as clubfoot, cleft palate, cystic ear swelling, and clinodactyly are present at birth in approximately 50% of individuals [16]. *COL9*-MED is generally the mildest form of MED and is characterized by joint pain in the hips and/or knees and stiffness presenting in the first decade of life, while radiographic abnormalities are primarily restricted to the knees with relative sparing of the hips [4]. Epiphyseal flattening and irregularity of affected joint are evident. MED-related myopathy has been reported in some families with *COL9A3*, *COMP*, and *COL9A2* mutations [17, 18].

We presently describe an autosomal dominant MED family with novel *COL9A3* mutation. The proband and his father presented with mild knee joint pain. The height of the proband was in the 20th percentile but his father’s height was average for Korean males. Radiographs of the proband showed epiphyseal changes of MED. The knee joints were most severely affected but other joints revealed minor involvement including ankle, foot, and wrist. Sparing of hip joints, no history of clubfoot or other deformities at birth, and the pattern of inheritance distinguished their conditions from the other MED caused by *COMP*, *MATN3*, and *DTDST* mutations. Therefore, we analyzed the genes associated with collagen type IX on the bases of clinical and radiographic phenotypes. It is interesting that the *COL9A3* mutation detected in the proband and his father is a missense mutation. Previously reported *COL9A3* mutations were splice site acceptor or donor mutations in intron 2 or 3, that invariably resulted in skipping of exon 3 of *COL9A3*[17, 19] with deletion of 12 amino acids in the COL3 domain of COL9A3. The c.104G> A substitution in exon 2 of *COL9A3*, resulting in p.Gly35Asp, affects the COL3 domain of COL9A3, and results in a substitution of Gly to Asp in the third position of “Pro-Pro-Gly” consecutive sequence. The COL3 domain is consistently affected in *COL9A2* and *COL9A3* mutations associated with MED, thus emphasizing the role of the COL3 domain in the pathogenesis of MED. Although, the novel mutation does not result in a splicing defect of the COL3 domain, it affects an important amino acid in a conserved “Pro-Pro-Gly” repeat sequence of the highly conserved COL3 domain.

Another point of interest is that the proband had been treated for seizure disorder. A study characterizing a microdeletion of 20q13.33, a cytogenetic locus in which *COL9A3* is located, demonstrated that this abnormality is associated with several clinical features including mental retardation, developmental delay, speech and language deficits, behavior problems and seizures. In the study, two of six patients had deletions that encompassed *COL9A3* and one had seizure disorder, but they lacked in clinical features reminiscent of MED. The association between seizure and *COL9A3* is not clear, but the possibility should be considered. In addition, the authors remarked that the suggested deletion of *COL9A3* did not cause the MED symptom [20]. However, we have a reservation with this opinion, because symptoms of MED caused by *COL9A3* mutations are mild and the patients with microdeletion at 20q13.33 had combined abnormalities in morphology and psychomotor and behavioral development. It should be defined after careful clinical and radiographic assessment.

Type IX collagen is the structural component of hyaline cartilage and vitreous of the eye, and has been observed in various tissues including the notochord, inner ear, heart, brain, and skin in mice [21]. Collagen IX is a hetero-trimeric molecule that consists of three α chains, α1(IX), α2(IX), and α3(IX)) in a 1:1:1 ratio. And the sizes of the domains and amino acid sequences of the α1(IX), α2(IX), and α3(IX) chains are highly conserved. Also the reported mutations in *COL9A2* and *COL9A3* show a remarkable consistency in affecting the COL3 domain [22], and clearly indicates that the COL3 domain is a functionally important component of type IX collagen in vivo. MD simulation of heterotrimer collagen IX revealed that the wild type structure formed hydrogen bonds with its neighboring strand, while the mutant showed self-aggregation. In addition, the distances between the mutation site (C-alpha) of wild type and mutant collagen are almost twice and the calculated energy state of the mutant is much lower, causing a self-aggregation. Our results reveal that the substitution of Gly to Asp produces a large local disruption to the COL3 domain of COL9A3, enough to lead to a malfunction of the type IX collagen.

Lastly, the phenotypic difference within family members was also observed but neither of the family members showed neuromuscular manifestations, a feature that has been associated in a previous MED patient with *COL9A3* mutation [17]. It remains to be determined whether both inter- and intra-familial phenotypic diversity are common in type IX collagen mutations. As well, the relationship between MED and other neuromuscular manifestations must be clarified.

## Consent

Written informed consent was obtained from the parents of the child for publication of this Case report and any accompanying images. A copy of the written consent is available for review by the Editor of this journal.

## Authors’ original submitted files for images

Below are the links to the authors’ original submitted files for images. Authors’ original file for figure 1 Authors’ original file for figure 2 Authors’ original file for figure 3
