# Supplementary figures and images for: Novel COL9A3 mutation in a family diagnosed with multiple epiphyseal dysplasia: a case report
Source: BMC Musculoskelet Disord. 2014 Nov 8;15:371. doi: 10.1186/1471-2474-15-371 (PMC4236474; doi:10.1186/1471-2474-15-371)

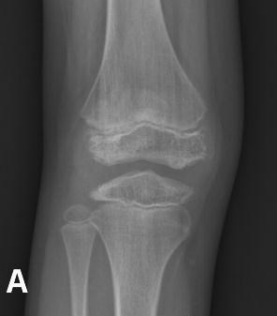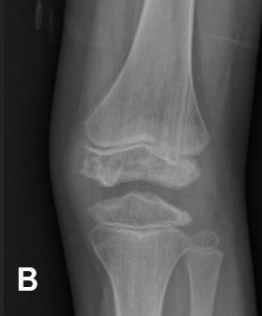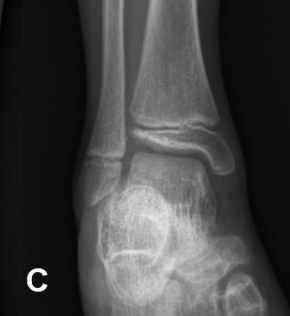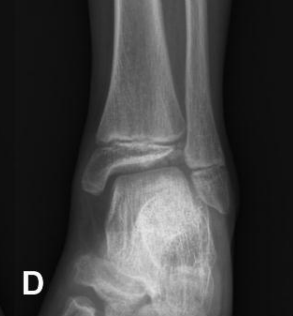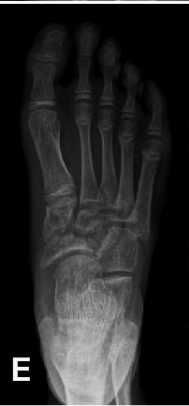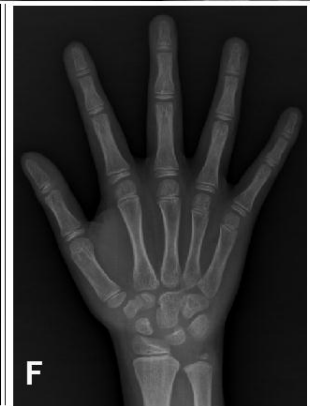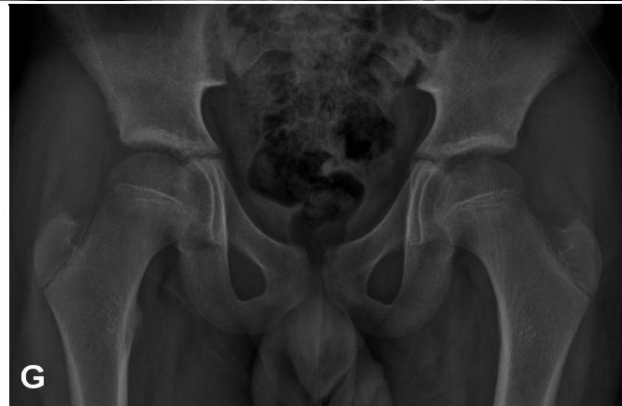

Supplement: Supplementary file 1 — Authors’ original file for figure 1 [file 12891_2014_2307_MOESM1_ESM.pdf]

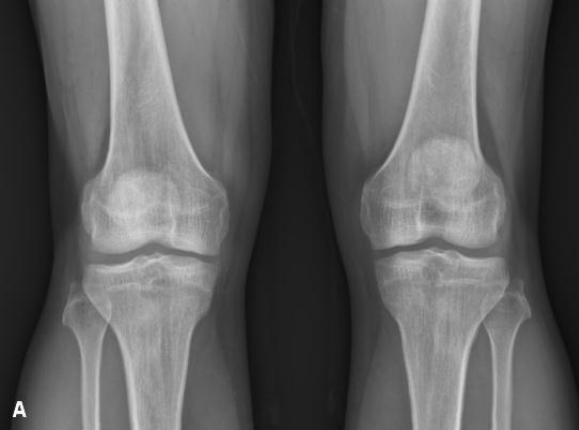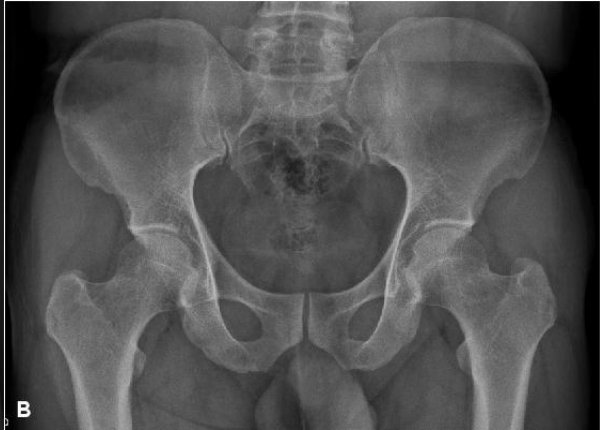

Supplement: Supplementary file 2 — Authors’ original file for figure 2 [file 12891_2014_2307_MOESM2_ESM.pdf]

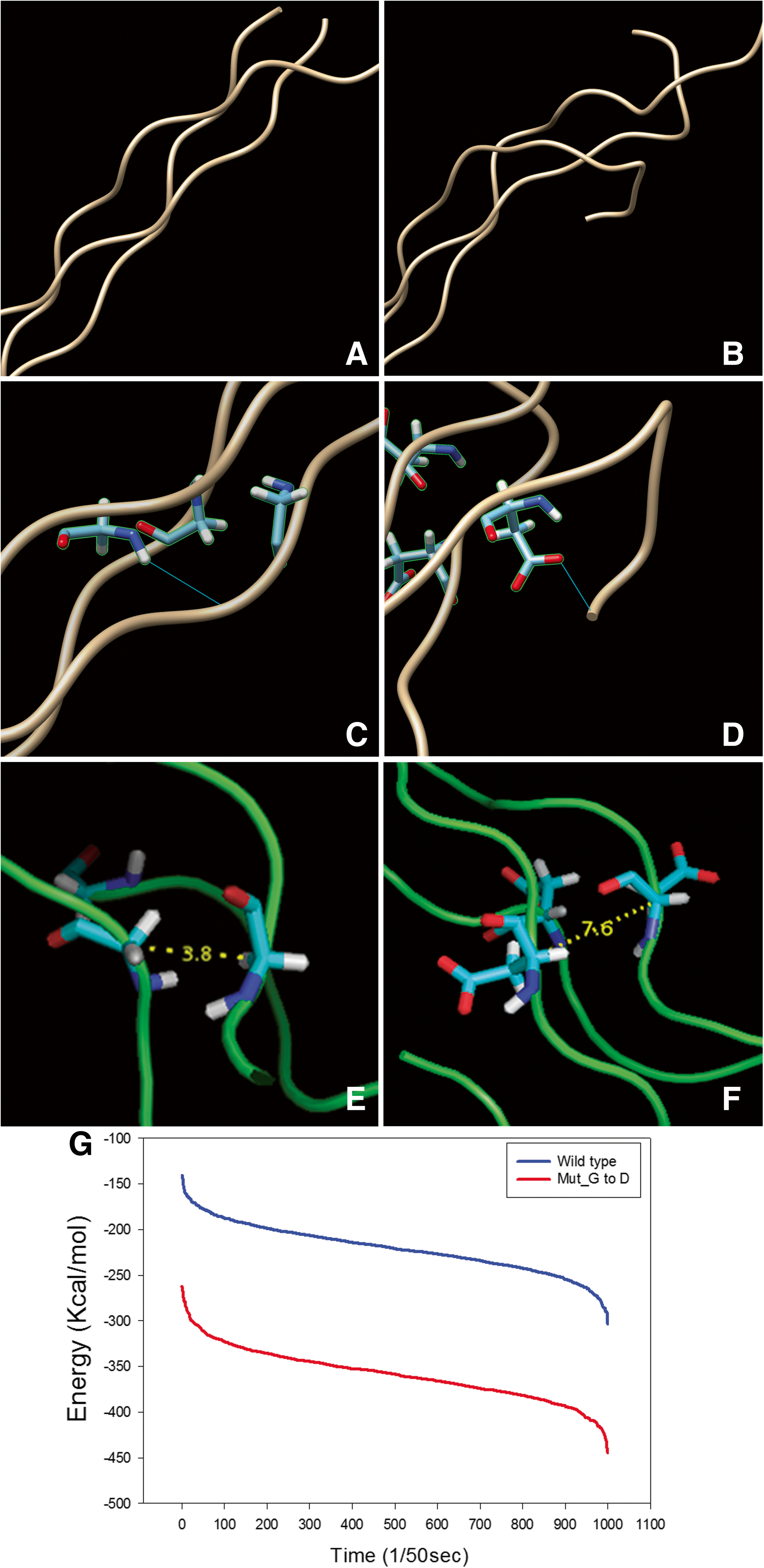

Supplement: Supplementary file 3 — Authors’ original file for figure 3 [file 12891_2014_2307_MOESM3_ESM.tiff]
